# Supplementary material for: Client satisfaction with pharmacy services and associated factors at Yekatit 12 Hospital Medical College, Addis Ababa, Ethiopia
Source: PLoS One. 2026 May 22;21(5):e0349245. doi: 10.1371/journal.pone.0349245 (PMC13196984; doi:10.1371/journal.pone.0349245)
Supplement: S1 File — (DOCX) [file pone.0349245.s001.docx]

Supplementary File 1: Questionnaire

ENGLISH VERSION OF QUESTIONNAIRE

Part 1:- Socio demographic characteristics of the study participants.

| No | Variable | Question |  |
| --- | --- | --- | --- |
| 101 | Gender | 1. Male 2. female |  |
| 102 | Age | 1. 18-25 2. 26-35 3. 36-50 4. Above 50 |  |
| 103 | Place of residence | 1. Urban 2. Rural |  |
| 104 | Marital status | 1. Single 2. Married 3. Divorce 4. Widowed |  |
| 105 | Religion | 1. Orthodox 2. Muslim 3. Protestant 4. Other |  |
| 106 | Education status | 1. No formal education 2. Primary education 3. Secondary education 4. Certificate and above |  |
| 107 | Occupation | 1. No job 2. Government employee 3. Farmer 4. House wife 5. Marchant 6. Daily laborer |  |
| 108 | wealth | 1. Income |  |
|  | Region | 1. Addis ababa 2. Afar 3. Amhara 4. Benishangul-gumuz 5. Dire dawa 6. Gambela 7. Harari 8. Oromia 9. Sidama 10. Somali 11. Southern Nations, Nationalities, and Peoples' Region (SNNPR) 12. Tigray |  |

**Part 2:- Patient experiences with pharmacy services in yekatit 12 hospital, Addis Ababa,**

**Ethiopia, 2025**

| No | Variable | Question |  |
| --- | --- | --- | --- |
| 201 | Familiarity with institution | 1. First visit 2. Chronic care |  |
| 202 | Self-judge health status | 1. Severely sick 2. Sick |  |
| 203 | Medication dispensed | 1. All 2. None or some |  |
| 204 | Payment modality | 1. Out-of-pocket 2. Paid by insurance 3. Free |  |
| 205 | Waiting time | 1. <15min 2. >15min |  |
| 205 | Patients’ views on the requirement to improve the service | 1. Improve medication availability 2. Increase waiting area space 3. Increase the number of staff 4. Reduce bureaucracy 5. Reduce waiting time |  |

**Part 3:- Study participants’ opinions towards the pharmacy setting, medication availability, and cost,**

| No |  | Very Dissatisfied | Dissatisfied | Neutral | Satisfied | Very Satisfied |
| --- | --- | --- | --- | --- | --- | --- |
| 301 | The pharmacy location is convenient |  |  |  |  |  |
| 302 | The private counseling area is comfortable and convenient |  |  |  |  |  |
| 303 | The pharmacy is clean |  |  |  |  |  |
| 304 | The waiting area is comfortable and convenient |  |  |  |  |  |
| 305 | The dispensary is clean |  |  |  |  |  |
| 306 | Medications I need are available |  |  |  |  |  |
| 307 | The cost of the medication is fair |  |  |  |  |  |
| 308 | The staff numbers are enough to the service |  |  |  |  |  |

**Part 4:- Study participants’ satisfaction towards the pharmacist approach or communication**

| No |  | Very Dissatisfied | Dissatisfied | neutral | Satisfied | Very Satisfied |
| --- | --- | --- | --- | --- | --- | --- |
| 401 | The politeness and interest of pharmacist was good |  |  |  |  |  |
| 402 | Pharmacists provide service equally |  |  |  |  |  |
| 403 | Pharmacists treat the client with dignity and respect |  |  |  |  |  |
| 404 | Pharmacy professionals were available during visit |  |  |  |  |  |
| 405 | The voice and tone of the pharmacy personnel was clear |  |  |  |  |  |
| 406 | Service waiting time in the pharmacy was fair |  |  |  |  |  |

**Part 5:- Study participants’ satisfaction towards the pharmacist medication instructions.**

| No |  | Very Dissatisfied | Dissatisfied | neutral | Satisfied | Very Satisfied |
| --- | --- | --- | --- | --- | --- | --- |
| 501 | Counselling time was enough |  |  |  |  |  |
| 502 | The pharmacy ensures that medications are taken as prescribed |  |  |  |  |  |
| 503 | Give administration instructions in understandable language |  |  |  |  |  |
| 504 | The pharmacist told me about proper storage of medications |  |  |  |  |  |
| 505 | Pharmacist tell about medication precautions and side effects |  |  |  |  |  |
| 506 | Medication and drug-drug and drug food interaction |  |  |  |  |  |
| 507 | Label readable and understandable instruction |  |  |  |  |  |
