# Supplementary material for: Client satisfaction with pharmacy services and associated factors at Yekatit 12 Hospital Medical College, Addis Ababa, Ethiopia
Source: PLoS One. 2026 May 22;21(5):e0349245. doi: 10.1371/journal.pone.0349245 (PMC13196984; doi:10.1371/journal.pone.0349245)
Supplement: Attachment — Submitted filename: Point by point response.docx [file pone.0349245.s005.docx]

**Point-by-Point Response to Reviewers**

We sincerely thank the reviewers for their valuable time, thoughtful feedback, and insightful comments on our manuscript. The suggestions have been extremely helpful in improving the overall quality, clarity, and academic rigor of our work. We have carefully considered each comment and revised the manuscript accordingly. Below, we provide a detailed, point-by-point response to each comment, explaining the modifications made and indicating where the corresponding changes can be found in the revised version.

**Reviewer 1**

**Title**

Q1: “Client satisfaction” is understandable, but in academic and professional writing, alternatives such as patient or customer satisfaction are considered more precise and widely accepted.

R1: Thank you for your comment. In our study, the respondents included both patients who directly received pharmacy services and caregivers who collected medications on behalf of patients, particularly for pediatric or bedridden patients. Therefore, we have retained the term “client satisfaction” as it more accurately represents both groups. However, we have clarified this definition in the methods section to avoid confusion and to align with professional standards. Please see at Page 7 line 19 to 27.

Q2: Who were your clients? Were these patients or their caregivers?

R2: In our study, clients included both patients and caregivers who directly received pharmacy services or collected medications on behalf of patients. For example, caregivers often collected medications for pediatric or bedridden patients who were unable to visit the pharmacy themselves. This clarification has now been added to the methods section of the revised manuscript to clearly define the study population. Please see at Page 7 line 19 to 27

Q3: Please mention the ethical approval offering body in abstract if you are writing that approval was obtained.

R3: We thank the reviewer for this important suggestion. Ethical approval for this study was obtained from the Institutional Review Board (IRB) of Yekatit 12 Hospital Medical College (Reference number: RPO/934/25). We have now included this information in the abstract.

Q4: Grammatical mistakes are present throughout the manuscript, revise it for language check.

R4: Apologies for the grammatical errors. The manuscript has been thoroughly revised and edited for grammar and clarity to improve readability.

Q5: Can you please write some detail on your healthcare system? Pharmacists were clinical pharmacist or hospital pharmacist? What was their job description hospitals?

R5: We thank the reviewer for this important comment. Yekatit 12 Hospital Medical College is a public referral hospital, providing a broad range of specialised healthcare services and serving as a teaching hospital in Addis Ababa, Ethiopia. The hospital pharmacy is staffed by hospital pharmacists who are responsible for dispensing medications, providing medication counselling, ensuring proper storage and handling of drugs, checking prescription appropriateness, and supporting clinical services in collaboration with other healthcare professionals. In the revised manuscript, we have added these details in the *Study Setting* and *Methods* sections to provide a clearer context of the healthcare system and the roles of pharmacists.

Q6: Can you provide the source of information regarding the hospital that you mentioned in your study setting to improve the reliability of data?

R6: We thank the reviewer for this suggestion. The information regarding Yekatit 12 Hospital was obtained directly from the hospital administration by request. This has now been clarified in the *Study Setting* section of the revised manuscript to ensure transparency and reliability of the data presented.

Q7: In methodology section, you mentioned that all patients were taken as clients. Should not you also include caregivers that generally approach pharmacies for medicines for in- patients? Or explain a bit about your hospital system in which how in-patients or bed- ridden patients can visit pharmacy?

R7: Thank you very much for your feedback. Both patients and caregivers who visited the pharmacy were included as study participants, as caregivers often collect medications on behalf of in-patients who cannot personally access the pharmacy. We acknowledge that this was not explicitly stated in the Methods section, and we have now revised the manuscript to clearly indicate that caregivers were included in the study population.

Q8: If you were just dealing with the patients, should not it be patient satisfaction instead of client satisfaction?

R8: As we included both patients and caregivers who received pharmacy services, client satisfaction would be appropriate terminology. To ensure clarity, we have now revised the Methods section to explicitly state that both patients and caregivers were included as participants.

Q9: In exclusion criteria, you have mentioned that clients below 18 years were excluded, while in abstract and results you have written that client who visited pediatric pharmacy, were significantly unsatisfied. It’s a bit confusing. Pediatric patients are above 18 years of age? How you have taken data from pediatric patients (below 18) who visited pediatric pharmacy?

R9: We sincerely thank the reviewer for this important clarification. In our study, participants were adult clients (aged 18 years and above) who received pharmacy services, including caregivers who collected medications from the pediatric pharmacy on behalf of children. Thus, no pediatric patients (below 18 years) directly participated in the study. The results referring to the pediatric pharmacy reflect the experiences and satisfaction of caregivers who obtained medicines for pediatric patients, not the children themselves. We have now clarified this point in the methods sections to avoid any confusion.

Q10: What if any patient refused to response, how randomization was maintained?

R10: Thank you for this comment. If a selected participant was unable to participate, the next available eligible participant would have been approached to maintain the sampling sequence. However, in our study, no selected participants refused to respond. This procedure ensured that the target sample size was achieved while maintaining representativeness.

Q11: For quantitative studies, generally structured tools are used for data collection. Why you used semi-structured questionnaire?

R11: Apologies for the typo. We have used a structured questionnaires in our study. The semi-structured term in the manuscript was an oversight, and we have corrected it to reflect that a structured questionnaire was used.

Q12: How you validated the study tool?

R12: The structured questionnaire was developed based on previously validated tools from relevant literature. Its content validity was ensured by adapting standard questions relevant to patient and caregiver satisfaction in pharmacy services. The questionnaire was widely used to measure client satisfaction for pharmacy services in Ethiopian setting (1, 2).

Q13: You did not explain about the scoring assumptions of your tool.

R13: We thank the reviewer for the comment. Satisfaction items were measured using a five-point Likert scale. For analysis, the mean score of all satisfaction items was calculated, and participants with scores above the mean were considered “Satisfied,” while those with scores below the mean were considered “Dissatisfied.” This approach allowed us to quantify overall satisfaction and maintain consistency across the study. We have clarified this in the manuscript for better understanding.

Q14: Discussion can be made rich while not only discussing your findings, but also providing global context.

R14: We appreciate the reviewer’s suggestion. We have revised the discussion section and compared our results with similar studies conducted globally.

Q15: In study strengths you are stating that “the relatively large sample size and use of a standardised, structured questionnaire also enhanced the reliability and consistency of the data collected: while above you have mentioned that you used semi-structured tool. It is confusing.

R15: Sorry for the confusion. We have corrected the semi-structured questionnaire typo error.

**Reviewer 2**

**Overall Assessment**

This manuscript addresses an important aspect of healthcare quality. The study is timely and relevant, with an adequate sample size and appropriate statistical methods. However, several methodological, interpretive, and reporting issues need clarification and revision before the paper can be considered for publication.

**Response:** We thank the reviewer for the thoughtful and constructive assessment of our manuscript. We appreciate the recognition of the study’s relevance, adequacy of the sample size, and appropriateness of the statistical methods. In the revised manuscript, we have carefully addressed each point raised by the reviewer.

**Major Comments**

**Clarity of Outcome Measures**

The manuscript reports a mean satisfaction score of 69.92 and a prevalence of 49.6% satisfied clients. These two metrics are conflated in both the abstract and main text, leading to confusion. Please clearly distinguish between the mean score and the proportion satisfied. Also, ensure consistency across the abstract, results, and discussion.

**Response:** We have revised the manuscript to clearly distinguish between the mean satisfaction score and the proportion of satisfied clients, and to ensure consistency across the abstract, results, and discussion sections.

**“Results**: A total of 49.76% of participants were satisfied with outpatient pharmaceutical services. Key factors significantly associated with satisfaction included residency, medication dispensed, and pharmacy type. Multivariable analysis showed urban residents were dissatisfied than rural residents (AOR = 0.36; 95% CI: 0.15–0.90; p = 0.015).” See abstract section.

**Operational Definition of Satisfaction**

Satisfaction was dichotomized using the mean score as the cut-off. This approach is arbitrary and may bias classification. Please justify the use of the mean and SD as a threshold and, if possible, provide references to support this method. Alternatively, consider using validated satisfaction indices or reporting results by Likert scale categories without collapsing them.

**Response:** We have revised the manuscript to provide a clearer justification for this approach. Specifically, we now explain that the use of the mean (or mean ± SD) as a cut-off has been employed in previous patient satisfaction studies where standardised or validated thresholds were not available. We have added references to support this methodological choice (3-5).

“The outcome variable, patient satisfaction, was categorised into “satisfied” and “unsatisfied” based on the mean satisfaction score calculated from 21 indicators (3). Patients scoring above the mean satisfaction score were classified as satisfied.”

**Data Presentation**

There are inconsistencies. For example, Table 1 reports *n = 408*, while the total sample is *422*. Please clarify reasons for missing responses and ensure consistent reporting. Figures are difficult to interpret, with overlapping labels and percentages. Simplify graphics, separate satisfaction vs dissatisfaction, and add clear legends (e.g., expand abbreviations such as ART, OPD).

**Response**: Thank you for your feedback. The correct sample size is 422 and we have revised the error accordingly. We removed Figure 1 and expressed the finding with text, as it was difficult to interpret.

**Interpretation of Results**

The explanation that rural participants report higher satisfaction due to “lower expectations” is speculative and not empirically supported. I would like to see some justification for this claim. Similarly, the lower satisfaction in emergencies and pediatric pharmacies is noted but not adequately explained. Please provide stronger evidence-based interpretations, referencing health system constraints or service delivery challenges in Ethiopia.

**Response:** Thank you for this insightful comment. We agree that the initial explanation regarding rural participants’ higher satisfaction being related to “lower expectations” was speculative and insufficiently supported by empirical evidence. After receiving your feedback, we re-examined the data and conducted an additional analysis to verify the association between residence and satisfaction. In the re-analysis, residence was no longer statistically significant. we removed it from the discussion section.

Regarding emergency and pediatric pharmacy settings, we have revised it as follows.

**“**Antiretroviral Therapy (ART) pharmacy users reported higher satisfaction compared to those in general outpatient pharmacies. In a study conducted in Dembia District, Ethiopia, patients attending ART pharmacy services exhibited a satisfaction rate of 75% (6). This might be due to ART services have shorter waiting times, stronger provider-patient engagement, and tailored counselling, which may increase patient satisfaction (7, 8). On the other hand, the emergency pharmacy users were less likely to be satisfied. This was consistent with a study conducted in Hawassa (9). The possible reason for this might be low availability of medication due to stock status of the pharmaceutical fund and supply agencies, low budget allocation to the hospital pharmacy(10).**”**

The regression tables show COR and AOR with CIs and p-values, but there is no mention of Model fit tests (e.g., Hosmer–Lemeshow, pseudo-R²). Checks for multicollinearity (education, income, and residence could be correlated). How confounders were selected for inclusion (all variables? those with p<0.2 in bivariable analysis?).

**Response**: Thank you very much for your feedback. We noted that the education variable demonstrated moderate multicollinearity in the initial model (maximum VIF = 5.7) and that the Hosmer–Lemeshow test indicated poor model calibration (p = 0.0127). In response, we recoded the education variable into three categories (“No education”, “Primary” and “Secondary and higher”). This adjustment reduced multicollinearity (maximum VIF = 4.4) and improved model fit, with the Hosmer–Lemeshow test becoming non-significant (p = 0.45), indicating adequate calibration. We appreciate your valuable comment.

We have also included the following statement in the methods section.

“Model adequacy was evaluated using the Hosmer–Lemeshow goodness-of-fit test and assessments of multicollinearity among predictor variables. The Hosmer–Lemeshow test was applied to examine the calibration of the logistic regression model by comparing observed and expected outcomes across deciles of predicted risk. A non-significant p-value was considered indicative of acceptable model fit. Multicollinearity was assessed using variance inflation factors (VIFs), with values greater than 10 regarded as evidence of problematic collinearity. Prior to final model selection, categorical predictors exhibiting moderate multicollinearity were recoded to ensure model stability and interpretability. The final model demonstrated satisfactory calibration and acceptable VIF values, confirming that the assumptions of logistic regression were met.”

For some predictors (e.g., religion “Other”), the CI is extremely wide (AOR 0.74; 95% CI: 0.07–7.54). This indicates small cell counts and unstable estimates. Similarly, pediatric and emergency subgroups may have small n, leading to inflated ORs and wide CIs.

**Response**: Thank you for raising this important point. We agree that the wide confidence interval observed for the religion category “Other” reflects very small cell counts and therefore produces an unstable estimate. Because several categories within the religion variable had sparse data, collapsing the variable into fewer groups was not feasible without losing conceptual meaning, and coding religion as a binary variable (e.g., “Orthodox” vs. “Other”) would not have been analytically appropriate given the heterogeneity across groups. Similarly, the wider confidence intervals observed for the paediatric and emergency pharmacy service groups reflect relatively small subgroup sizes.

In the abstract and discussion, the authors sometimes phrase ORs as “urban residents were dissatisfied” instead of “less likely to be satisfied.” This could confuse readers. Also, the ART pharmacy AOR = 2.02 (95% CI: 0.91–4.50) is not significant, but in the discussion it’s presented as a positive finding.

**Response**: Thank you, we have revised and corrected it accordingly.

**“**A total of 49.76% of participants were satisfied with outpatient pharmaceutical services. Multivariable analysis showed participants with secondary and above were less likely to be satisfied than those with no education (AOR = 0.23; 95% CI: 0.07,0.79; p = 0.020). Clients who did not receive all prescribed medications were less likely to be satisfied (AOR = 0.35; 95% CI: 0.20,0.60; p = 0.000). ART pharmacy users were more likely to be satisfied compared to outpatient pharmacy (AOR = 3.27; 95% CI: 1.38,7.78; p = 0.007).**”**

**Discussion and Literature Review**

The introduction and discussion contain repetition and limited engagement with recent global or regional systematic reviews on patient satisfaction. Strengthen the literature review by presenting your findings in the broader context of pharmacy service quality frameworks.

**Response:** Thank you very much for this comment. We have substantially strengthened the literature review in both introduction and discussion sections.

**Limitations**

Important limitations are under-reported. To report a few are

a) Potential interviewer bias (interviews conducted within hospital premises), non-response bias (even though 10% adjustment was made), Reliance on self-reported satisfaction without triangulation (e.g., service audits).

**Response:** We agree with the reviewer that important limitations were under-reported in the initial submission. In response, we have expanded the limitations section to address these issues.

“Additionally, since the study was conducted at a single hospital, the findings may not be generalizable to other settings. There is also a potential for interviewer bias or social desirability bias, as interviews were conducted within the hospital premises. Moreover, the study did not assess other potentially influential factors, such as prior experiences with pharmacy services or patient expectations, which may have impacted satisfaction responses. Additionally, as the study relies on self-reported satisfaction may limit objectivity, as no triangulating data were collected.”

**Language and Style**

The manuscript requires careful language editing. A language editor is suggested.

**Response:** We have thoroughly revised the manuscript to improve clarity, grammar, and overall readability. Additionally, the revised version has undergone careful language editing to ensure that the presentation meets the required academic standards.

**Minor Comments**

**References**: Ensure all references follow PLOS ONE formatting guidelines. Some are abbreviated inconsistently.

**Response:** We have carefully reviewed all references and revised them to fully comply with the PLOS ONE formatting guidelines.

**Author Information**: I am curious, is this a real email ID ([iloveyousomuhe@gmail.com](mailto:iloveyousomuhe@gmail.com))? This appear unprofessional. Consider using institutional or professional addresses.

Response: Unfortunately, the author does not have institutional email address. However, we have used another email address.

**Figures/Tables**: Add clear legends to explain abbreviations and variables used.

Response: We have used clear legends and explain abbreviations.

**Abstract**: Avoid repetition and ensure that results are presented in a concise, non-interpretive manner.

Response: We have revised the abstract to avoid repetition and to ensure that the results are presented concisely and without interpretation.

**References**

1.	Molla M, Sisay W, Andargie Y, Kefale B, Singh P. Patients’ satisfaction with outpatient pharmacy services and associated factors in Debre Tabor comprehensive specialized hospital, Northwest Ethiopia: A cross-sectional study. PLoS One. 2022;17(1):e0262300.

2.	Semegn S, Alemkere G. Assessment of client satisfaction with pharmacist services at outpatient pharmacy of Tikur Anbessa Specialized Hospital. PLOS ONE. 2019;14(10):e0224400.

3.	Berehe TT, Bekele GE, Yimer YS, Lozza TZ. Assessment of clients satisfaction with outpatient services at Yekatit 12 Hospital Medical College, Addis Ababa, Ethiopia. BMC Research Notes. 2018;11(1):507.

4.	Ayele Y, Hawulte B, Feto T, Basker GV, Bacha YD. Assessment of patient satisfaction with pharmacy service and associated factors in public hospitals, Eastern Ethiopia. SAGE Open Med. 2020;8:2050312120922659.

5.	Surur AS, Teni FS, Girmay G, Moges E, Tesfa M, Abraha M. Satisfaction of clients with the services of an outpatient pharmacy at a university hospital in northwestern Ethiopia: a cross-sectional study. BMC Health Services Research. 2015;15(1):229.

6.	Belete TM, Tadesse SA, Atnafu K, Kelemu M, Asrie ABJAR, Therapy. Patient satisfaction with antiretroviral therapy service provided by pharmacists in Dembia district health institutions, Northwest Ethiopia. 2023;20(1):38.

7.	Katongole SP, Mukama SC, Nakawesi J, Bindeeba D, Simons E, Mugisa A, et al. Enhancing HIV treatment and support: a qualitative inquiry into client and healthcare provider perspectives on differential service delivery models in Uganda. AIDS Res Ther. 2024;21(1):47.

8.	Jabessa M, W.Daka D, Ejigu Y, Tolessa F, Fufa D. Patient Satisfaction with Antiretroviral Therapy Service and Associated Factors at Jimma Town Public Health Facilities, Southwest, Ethiopia. HIV/AIDS - Research and Palliative Care. 2021;13:691-7.

9.	Worku M, Loha E. Assessment of client satisfaction on emergency department services in Hawassa University Referral Hospital, Hawassa, Southern Ethiopia. BMC Emerg Med. 2017;17(1):21.

10.	Berassa MS, Worku SG. Availability of Essential Medicines at a University Hospital in Addis Ababa, a cross-sectional study. Ethiopian Journal of Health Development. 2023;37(3):1-5.
