# Supplementary material for: Client satisfaction with pharmacy services and associated factors at Yekatit 12 Hospital Medical College, Addis Ababa, Ethiopia
Source: PLoS One. 2026 May 22;21(5):e0349245. doi: 10.1371/journal.pone.0349245 (PMC13196984; doi:10.1371/journal.pone.0349245)
Supplement: Attachment — Submitted filename: Response to Reviewers.docx [file pone.0349245.s006.docx]

**Point-by-Point Response to the Editor**

We sincerely thank the Editor for the careful review and valuable feedback. Please find our point-by-point responses below.

**Comment**: Please ensure that the references are formatted using the Vancouver style, as per the PLOS ONE submission guidelines https://journals.plos.org/plosone/s/submission-guidelines. In particular, please ensure that each citation in the reference list states the journal name.

**Response**: Thank you very much for the comment, and we apologise for overlooking the journal names in the reference list. We have now revised all references to ensure they follow Vancouver style and are fully consistent with the PLOS ONE submission guidelines, including the inclusion of journal names for each citation.
