# Supplementary material for: Client satisfaction with pharmacy services and associated factors at Yekatit 12 Hospital Medical College, Addis Ababa, Ethiopia
Source: PLoS One. 2026 May 22;21(5):e0349245. doi: 10.1371/journal.pone.0349245 (PMC13196984; doi:10.1371/journal.pone.0349245)
Supplement: Attachment — Submitted filename: Reviewer comments.docx [file pone.0349245.s002.docx]

Reviewer comments

This study highlighted the significance of assessing patient or customer satisfaction with pharmacy services as a key indicator of healthcare quality. It provided insights into how pharmacy practices impact patient trust, adherence and overall healthcare outcomes. However, I have comments that might improve the quality of manuscript.

1. “Client satisfaction” is understandable, but in academic and professional writing, alternatives such as patient or customer satisfaction are considered more precise and widely accepted.
2. Who were your clients? Were these patients or their caregivers?
3. Please mention the ethical approval offering body in abstract if you are writing that approval was obtained.
4. Grammatical mistakes are present throughout the manuscript, revise it for language check.
5. Can you please write some detail on your healthcare system? Pharmacists were clinical pharmacist or hospital pharmacist? What was their job description hospitals?
6. Can you provide the source of information regarding the hospital that you mentioned in your study setting to improve the reliability of data?
7. In methodology section, you mentioned that all patients were taken as clients. Should not you also include caregivers that generally approach pharmacies for medicines for in-patients? Or explain a bit about your hospital system in which how in-patients or bed-ridden patients can visit pharmacy?
8. If you were just dealing with the patients, should not it be patient satisfaction instead of client satisfaction?
9. In exclusion criteria, you have mentioned that clients below 18 years were excluded, while in abstract and results you have written that client who visited pediatric pharmacy, were significantly unsatisfied. It’s a bit confusing. Pediatric patients are above 18 years of age? How you have taken data from pediatric patients (below 18) who visited pediatric pharmacy?
10. What if any patient refused to response, how randomization was maintained?
11. For quantitative studies, generally structured tools are used for data collection. Why you used semi-structured questionnaire?
12. How you validated the study tool?
13. You did not explain about the scoring assumptions of your tool.
14. Discussion can be made rich while not only discussing your findings, but also providing global context.
15. In study strengths you are stating that “the relatively large sample size and use of a standardised, structured questionnaire also enhanced the reliability and consistency of the data collected: while above you have mentioned that you used semi-structured tool. It is confusing.
