# Supplementary material for: Client satisfaction with pharmacy services and associated factors at Yekatit 12 Hospital Medical College, Addis Ababa, Ethiopia
Source: PLoS One. 2026 May 22;21(5):e0349245. doi: 10.1371/journal.pone.0349245 (PMC13196984; doi:10.1371/journal.pone.0349245)
Supplement: Attachment — Submitted filename: Reviewer Comments PLos.docx [file pone.0349245.s003.docx]

**Reviewer Comments**

**Assessment of Client Satisfaction with Pharmacy Services and Associated Factors at Yekatit 12 Hospital Medical College, Addis Ababa, Ethiopia 2025**

**Overall Assessment**

This manuscript addresses an important aspect of healthcare quality. The study is timely and relevant, with adequate sample size and appropriate statistical methods. However, several methodological, interpretive, and reporting issues need clarification and revision before the paper can be considered for publication.

**Major Comments**

**Clarity of Outcome Measures**

The manuscript reports a mean satisfaction score of 69.92 and a prevalence of 49.6% satisfied clients. These two metrics are conflated in both the abstract and main text, leading to confusion. Please clearly distinguish between the mean score and the proportion satisfied. Also, ensure consistency across the abstract, results, and discussion.

**Operational Definition of Satisfaction**

Satisfaction was dichotomized using the mean score as the cut-off. This approach is arbitrary and may bias classification. Please justify the use of the mean and SD as a threshold and, if possible, provide references to support this method. Alternatively, consider using validated satisfaction indices or reporting results by Likert scale categories without collapsing them.

**Data Presentation**

There are inconsistencies. For example, Table 1 reports *n = 408*, while the total sample is *422*. Please clarify reasons for missing responses and ensure consistent reporting. Figures are difficult to interpret, with overlapping labels and percentages. Simplify graphics, separate satisfaction vs dissatisfaction, and add clear legends (e.g., expand abbreviations such as ART, OPD).

**Interpretation of Results**

The explanation that rural participants report higher satisfaction due to “lower expectations” is speculative and not empirically supported. I would like to see some justification for this claim. Similarly, the lower satisfaction in emergencies and pediatric pharmacies is noted but not adequately explained. Please provide stronger evidence-based interpretations, referencing health system constraints or service delivery challenges in Ethiopia.

The regression tables show COR and AOR with CIs and p-values, but there is no mention of Model fit tests (e.g., Hosmer–Lemeshow, pseudo-R²). Checks for multicollinearity (education, income, and residence could be correlated). How confounders were selected for inclusion (all variables? those with p<0.2 in bivariable analysis?).

For some predictors (e.g., religion “Other”), the CI is extremely wide (AOR 0.74; 95% CI: 0.07–7.54). This indicates small cell counts and unstable estimates. Similarly, pediatric and emergency subgroups may have small n, leading to inflated ORs and wide CIs.

In the abstract and discussion, the authors sometimes phrase ORs as “urban residents were dissatisfied” instead of “less likely to be satisfied.” This could confuse readers. Also, the ART pharmacy AOR = 2.02 (95% CI: 0.91–4.50) is not significant, but in the discussion it’s presented as a positive finding.

**Discussion and Literature Review**

The introduction and discussion contain repetition and limited engagement with recent global or regional systematic reviews on patient satisfaction. Strengthen the literature review by presenting your findings in the broader context of pharmacy service quality frameworks.

**Limitations**

Important limitations are under-reported. To report a few are

a) Potential interviewer bias (interviews conducted within hospital premises), non-response bias (even though 10% adjustment was made), Reliance on self-reported satisfaction without triangulation (e.g., service audits).

**Language and Style**

The manuscript requires careful language editing. A language editor is suggested.

**Minor Comments**

**References**: Ensure all references follow PLOS ONE formatting guidelines. Some are abbreviated inconsistently.

**Author Information**: I am curious, is this a real email ID ([iloveyousomuhe@gmail.com](mailto:iloveyousomuhe@gmail.com))? This appear unprofessional. Consider using institutional or professional addresses.

**Figures/Tables**: Add clear legends to explain abbreviations and variables used.

**Abstract**: Avoid repetition and ensure that results are presented in a concise, non-interpretive manner.
